# Palatal Diseases as Applied to Dentistry: Pathology and Cases

**Published:** 1893-10

**Authors:** Vida A. Latham

**Affiliations:** Chicago, Ill.


### PALATAL DISEASES AS APPLIED TO DENTISTRY: PATHOLOGY AND CASES.[1]

BY VIDA A. LATHAM, D.D.S., F.R.M.S., CHICAGO, ILL.

DISEASES of the palate are not so rare as is often thought by physicians and dental surgeons, if attention be paid to that part of the mouth.

The anatomical position of the palate, by its structural continuity with the alveoli and gums in front, and its connection posteriorly with the tongue and pharynx by means of the pillars of the fauces, is liable to implication by the extension of disease from these parts; whilst, on the other hand, morbid processes originating in the palate may spread to the neighboring portions of the mouth, fauces, or pharynx.

---

[1] Read before the World's Columbian Dental Congress, Chicago, August, 1893.

The physician sees many cases of tonsillitis, but seldom or never considers them further than the present needs of the case require.

The condition of the palato-glossal fold should always be studied in tonsillitis, more particularly in periadimitic changes.

Inflammation of the buccal tissue is not commonly found extending onto the mucous membrane covering the anterior edge of the coronoid process (the precoronoid space of Allen). Inflammatory action about the inferior third molar is readily transmitted to it, thence involving the soft palate. Conversely, ulcerations of the soft palate may tend to involve the tissues around the inferior third molar.

Another fold of great importance is the palato-pharyngeal. This determines the site of pharyngeal irritation, for immediately to the median side a corrugation of the pharyngeal mucous membrane is usually met with in pharyngitis, more particularly in syphilitic angina. In the seat of ulcerations the study of these folds is also of primary importance.

It is necessary to remember that the palate, with the posterior pillars of the fauces, constitutes a septum between the nasal and buccal passages, which by contraction of the palato-pharyngei muscle becomes complete in the act of deglutition, and thus cuts off communication between the two cavities. Therefore, its structural and functional integrity is essential for perfect articulation, suction, and deglutition. The chief symptoms caused by lesions of this part are usually dependent upon impairment of these physiological acts.

The diseases of the palate may be grouped into:

I. Congenital malformations, as cleft palate, which it is better to treat by itself as a subdivision of palatal disease.

II. Inflammation.

III. Ulcers.

IV. Necrosis; and

V. Tumors.

[Note.—The detailed description of the several classes of palatal diseases which follow will be found in the paper as published in the Congress transactions.]

Case I.—A girl, aged eight years, on examination showed a tumor of the palate, which was not apparently the cause of pain or difficulty in swallowing. On examining the growth more closely, it was found to be about the size of a large walnut, extending from the soft palate on the right side, and projecting downward towards the end of the tongue. It was unconnected with the bone, and had a broad attachment. The microscopical slides showed small round and spindle-shaped cells in great numbers, without any definite stroma.

Case II.—A lady, aged sixty-six, consulted a dentist with reference to the fit of her plate; on examining the mouth, a nodule was found occupying the right half of the soft palate and growing forward over the hard palate, inward and across the middle line, and downward so as to touch the tongue. It was globular, soft, and elastic, almost fluctuating; the mucous membrane over it was thin and tense, not adhesive, and there were no enlarged glands.

The growth had been developing two years, lately with more rapidity. The voice was thick and indistinct, but swallowing was easy and painless. At the operation the mucous membrane was incised and some of it stripped off; then the growth easily shelled out. It was not firmly attached, but lay loosely in the palate in a distinct capsule. Hemorrhage was free, though easily controlled by pressure. On microscopical examination I found the specimen to be a mixed round and spindle-celled sarcoma, with much embryonic connective tissue and some imperfectly-formed tabular glands.

Case III. was a tumor from the hard palate of a woman aged forty-five. This was fourteen years in duration. It was small, circumscribed, and freely movable; the mucous membrane over it being smooth and natural. The tumor was easily shelled out. In structure it had a fibrous ground substance, broken up by masses of cells into curious tracts and processes with sinuous, crescentic, and indented edges, sometimes split and frayed. The cell-masses were heterogeneous; many large squamous epithelial cells, also cell-nests. In and around the tracts of fibrous matrix were scattered shrunken, ill-shaped cells, which suggested that this matrix and the cell-nests may have been due to degeneration or perversion of masses of cells. In other parts of the section the cells were of glandular type, and laid down in tubules. The general aspect of this tumor under the microscope proved it to be altogether irregular and embryonic.

Case IV.—This case is of much interest on account of its rare character, for so far I can only find mention of two other cases observed in this locality. The patient, a woman aged fifty-three, blonde, excellent health, has been frequently to the dentist on account of loss of teeth in the lower jaw (the reason for losing them being indefinite). A partial plate was made, but it never fitted well, and had always been such a source of annoyance by causing irritation of the hard palate near the alveolus to the right of the median line that she removed the plate constantly.

About six months before she consulted another dentist a small soft swelling appeared upon the irritated part of the hard palate behind the alveoli of the incisors, smooth and painless. As the pain increased, she came to have the remaining tooth out on the right side, and for two months the plate could not be worn on account of the growth. The pain now having increased in severity, an operation was urged, to which she finally consented. The extent of the tumor was almost to the alveolus on the anterior and external sides, and to the middle line on the inner side; posteriorly to the suture between the maxilla and the palate-bone.

The growth was flat, sharply defined, a raised surface, and a rounded outline. It was uniformly firm, but not hard, of a purplish-black color, and occurring in patches. There were no enlarged glands.

The operation consisted in removing the whole right side of the hard palate, including the alveolus. To give room, the upper lip was freely detached from the bone.

The growth was quite firm and black on section, almost like blood. The bone was invaded with the growth, and in the central part was absorbed to the extent of a five-cent piece.

The patient made a good and rapid recovery. The face suffered no deformity. An obturator was adjusted to the palate, and the patient articulated quite clearly.

On making micro-sections, staining with logwood, methyl blue, etc., the tumor proved to be a spindle-celled sarcoma originating from the periosteum, as the examination clearly showed. The cells were large, with a considerable amount of intercellular tissue. No myeloid cells; mass unevenly pigmented. Pigment appeared in irregular blotches, brown in color, and deposits made up of granules of ruddy brown and others black in color. The cause was no doubt well localized and long-continued irritation.

It is well to note that pigment is not usually found in the human palate, but is common in many of the lower animals.

Case V.—Adenoma of the palate. The patient, aged fifty, had known of the growth for eighteen months. It passed back on the soft palate on the left side, ulcerating in the middle, and painless. The ulcer was a distinct feature in the case, and it was referred to a number of surgeons, who agreed on its being an exceptional case. A cheesy, fetid mass, such as is often obtained from tonsillar follicles, was removed from the bottom of the ulcer. In the operation the whole thickness of the soft palate had to be removed. Recovery was good. On micro-section it showed adenoma. The fibrous tissue was readily seen, and seemed to form an ill-marked capsule around the periphery of the tumor.

Case VI.—Patient, a laborer, aged sixty-five, excellent health, had a growth of one to one and a half inches in circumference, covered by healthy non-adherent mucous membrane on the left side of the soft palate. He had noticed the growth for eight months. For three months it began to increase slowly, then burst through the mucous membrane in front of the palate. The last two months it grew rapidly.

The extent was by the hard palate in front, by alveolus to the left, the middle line of the soft palate to the right, tonsils and hard palate normal. No glands enlarged, nor epistaxis. Some thirty years ago patient had a similar growth on same part of soft palate. The operation was very extensive, and was performed by Dr. G. M. Brennan, to whom I am indebted for the specimen. The left common carotid was ligated, and the growth with the whole of the affected side of the soft palate removed, partly by knife and partly by thermo-cautery.

The tumor was encapsulated, about the size of a large walnut, pinkish-gray in color, and was diagnosed as alveolar sarcoma. Recovery was good.

On section large connective-tissue cells were everywhere arranged in larger and smaller groups by closely-intersecting bands of well-formed fibrous tissue. The individual cells separated by delicate fibrils with larger trabeculæ.

Primary tumors of the soft palate seem very rare. From isolated reports it would appear that all forms of connective-tissue growths have been met with in this region. It has been the seat of adenoid cancer, fleshy growths vaguely called myoma, dermoid, and papillomatous tumors. So far I have only been able to find four accounts of primary tumors of the soft palate, a cancer, a papilloma, a dermoid, and an adenoma.

Case VII.—Myxo-sarcoma. This is another form of the sarcoma groups, and seems the variety most frequent in this region. The patient was a woman aged thirty, who had noticed the swelling five years ago. It increased gradually to within five months of the operation for its removal, then began to grow rapidly and with severe pain. It appeared as a distinctly circumscribed, smooth, slightly undulating, firm, and immovable growth, the size of a hen's egg, on the left side of the hard palate, and involving some of the anterior portion of the soft palate. There was a distinct egg-shell crackling at circumference, but none over its central part. The mucous membrane over it was healthy; no gland-enlargement. The tumor appears to spring from the periosteum, and contains a large cyst. It was diagnosed spindle-celled sarcoma, intermixed with mucus, and this was afterwards proved by microscopical examination.

As regards the treatment, I will simply mention a few points worthy of attention. The chief danger in palatal operations is from hemorrhage,—the ingress of blood into the trachea, especially when the patient is under ether or chloroform. Cocaine renders this danger much less, but gives another form of trouble by its uncertain action. The difficulties in differential diagnosis are many, and it requires much study to know the nature of the growths and to classify them properly, on account of their irregular morbid histology.

